# Oocyte and dietary supplements: a mini review

**DOI:** 10.3389/fcell.2025.1619758

**Published:** 2025-06-26

**Authors:** Hao Chen, Shuoqi Wang, Meiying Song, Dongxia Yang, Hongmei Li

**Affiliations:** ^1^ First Clinical Medical College, Heilongjiang University of Chinese Medicine, Harbin, China; ^2^ Department of Gynecology II, Second Affiliated Hospital of Heilongjiang University of Chinese Medicine, Harbin, China

**Keywords:** oocyte process, quality ovary, dietary supplements, lifestyle changes, clinical trials

## Abstract

Rising rates of infertility have stimulated interest in dietary supplements to improve oocyte quality through mitochondrial function, antioxidant activity, and epigenetically regulated metabolic pathways. Mitochondria provides adenosine triphosphate for oocyte maturation, with Coenzyme Q10 (CoQ10) demonstrating efficacy in animal models by alleviating oxidative damage and enhancing blastocyst formation. In aged mice, CoQ10 restored mitochondrial activity and reduced chromosomal abnormalities, while preliminary human studies noted improved embryo quality in poor responders, though randomized controlled trials (RCTs) remain inconclusive. Antioxidants like melatonin counter reactive oxygen species (ROS)-induced spindle defects and mitochondrial dysfunction, showing benefits in murine oocyte maturation and blastocyst development. Resveratrol enhanced bovine oocyte quality through metabolic modulation. Human trials on antioxidants show reduced granulosa cell stress but lack robust evidence. Epigenetically, folate supports DNA methylation critical for embryonic gene expression, with deficiencies linked to hyperhomocysteinemia and developmental defects in animal models. Human observational studies associate folate-rich diets with lower aneuploidy and better assisted reproductive technology outcomes, while omega-3 fatty acids aid chromatin remodeling via histone deacetylase regulation. Despite compelling preclinical data, human trials face inconsistencies due to variable designs and confounders. Standardized RCTs are urgently needed to translate mechanistic insights into clinical guidelines, addressing the disconnect between animal studies and human reproductive outcomes.

## 1 Introduction

Infertility is defined as the inability to conceive after 1 year of unprotected sexual intercourse. While male factors, such as low sperm count, poor sperm motility, or hormonal imbalances, contribute to this condition, reduced female reproductive capacity remains a critical determinant (Hosseini et al., 2024). Poor female fertility is frequently associated with clinical features such as elevated body mass index (BMI), advanced maternal age, and polycystic ovary syndrome (PCOS). For instance, PCOS is characterized by hormonal imbalances, including elevated anti-Müllerian hormone (AMH), luteinizing hormone (LH), and androgen levels, alongside reduced follicle-stimulating hormone (FSH), which impair ovulation and fertility (Zabieglo et al., 2025). Furthermore, age-related declines in ovarian reserve and BMI-related metabolic disturbances, such as increased free androgen index (FAI) and decreased sex hormone-binding globulin (SHBG), are also significant contributors to fertility challenges.

*In vitro* fertilization (IVF) has emerged as a leading assisted reproductive technology (ART) for overcoming infertility, offering hope to millions of couples worldwide. However, the high costs associated with IVF treatments, coupled with their emotional and psychological toll, pose significant challenges. Couples often spend an average of $61,377 out-of-pocket across 2.7 cycles to achieve a live birth, placing substantial financial and emotional strain on individuals and families (Peterson et al., 2025). This burden is further exacerbated by the uncertain outcomes of IVF procedures, which can lead to repeated cycles and additional costs. In light of these challenges, optimizing the efficacy of IVF protocols, including the use of dietary supplements, becomes even more critical to reduce the overall economic and psychological toll on patients.

Although pharmacological therapies retain their efficacy, dietary supplements, including coenzyme Q10 (CoQ10), myo-inositol, melatonin, and vitamins, have emerged as a promising adjunct in infertility management due to their potential to mitigate oxidative stress, improve hormonal balance, and enhance reproductive capacity (Vitagliano et al., 2021). However, despite accumulating evidence, inconsistencies in findings related to macromolecule and nutrient intake highlight the need for clearer mechanistic insights. For example, CoQ10 has demonstrated efficacy in improving oocyte quality and clinical pregnancy rates, particularly in the context of ovarian aging (Shang et al., 2024). Similarly, other supplements such as myo-inositol, melatonin, and vitamins have also show significant benefits in IVF outcomes. Myo-inositol, for example, enhances embryo quality and reduces unsuitable oocytes under optimal drug stimulation (Zheng et al., 2017; Wdowiak et al., 2025). Notably, myo-inositol is a key molecule in FSH signaling and oocyte maturation, and its supplementation has been shown to improve the metaphase II (MII) oocyte rate and fertilization rate, particularly in women with PCOS and non-obese PCOS (Zhang et al., 2025). Additionally, it reduces the amount of gonadotropins required for ovarian stimulation and shortens the stimulation length, especially in women with PCOS (Lagana et al., 2018). Emerging evidence also suggests that myo-inositol supplementation may improve ART outcomes in poor ovarian responders, evidenced by increased fertilization rates, elevated ovarian sensitivity index, and reduced gonadotropin demands (Mohammadi et al., 2021).

Furthermore, while serum mineral supplementation has been associated with improved oocyte quality, supports embryo development, mitigates oxidative stress, regulates hormonal balance, and optimizes IVF outcomes, while concurrently reducing miscarriage rates (Kapper et al., 2024). In patients with PCOS, vitamin D supplementation elevates ovulation and pregnancy rates, lowers androgen levels and miscarriage rates, and reduces FSH and LH concentrations, despite having no measurable impact on cleavage or fertilization rates (Yang et al., 2023). Astaxanthin, a potent antioxidant, enhances oocyte quality and reduces oxidative stress in ART procedures (Maleki-Hajiagha et al., 2024). Notably, astaxanthin improves total antioxidant capacity (TAC) in follicular fluid but exerts inconsistent effects on catalase (CAT), malondialdehyde (MDA), or superoxide dismutase (SOD) levels. While it moderately enhances oocyte and embryo quality, its impact on fertility rates remains statistically insignificant (Rodrigues et al., 2025). Oral melatonin administration during IVF cycles has been associated with increased mature oocytes yields and higher clinical pregnancy rates, although these improvements lack statistical significance (Mejlhede et al., 2021). However, melatonin supplementation during controlled ovarian stimulation, while linked to low rates of congenital abnormalities, shows no clinical benefits in terms of oocyte retrieval efficiency or miscarriage prevention (Seko et al., 2014). Mitochondrial supplementation, though safe, fails to improve oocyte competence, with studies reporting no significant changes in embryo development or fertilization rates (Ferreira et al., 2021).

Despite accumulating evidence, inconsistencies in findings related to macromolecule and nutrient intake highlight the need for clearer mechanistic insights. This review synthesizes current evidence on molecular pathways underlying infertility interventions and emphasizes priority directions for future research.

## 2 Mechanistic and biological pathways involving dietary supplements on oocytes

The rising rates of infertility have stimulated interest in dietary supplements to improve oocyte quality through mitochondrial function, antioxidant activity, and epigenetically regulated metabolic pathways. This section discusses the mechanisms and biological pathways of dietary supplements in improving oocyte quality, with a focus on their roles in different animal models and human studies.

### 2.1 Mitochondrial function

Mitochondrial dysfunction is a key factor in oocyte quality decline, and dietary supplements have shown promising effects in enhancing mitochondrial adenosine triphosphate (ATP) synthesis, reducing oxidative stress, and ultimately improving oocyte quality. Specifically, nicotinamide riboside (NR) supplementation during early embryonic development has been demonstrated to mitigate reactive oxygen species (ROS) accumulation, thereby preventing apoptosis and DNA damage in IVM mouse models. Although nicotinamide adenine dinucleotide (NAD+), a critical redox cofactor, declines in post-ovulatory oocytes, NR supplementation effectively prevents this loss, restoring metaphase II (MII) oocyte quality by maintaining chromosomal integrity and alleviating mitochondrial dysfunction, which may enhance ART success (Li H. et al., 2023). Notably, NAD + precursors also show therapeutic potential for ovarian infertility in a PCOS mouse model (Zhu et al., 2025). Furthermore, CoQ10 increases glucocorticoid receptor expression while reducing immunophilins (FK506-binding protein 5, FKBP5) and hydroxysteroid dehydrogenases (HSD11B1), thereby promoting oocyte maturation (Ruiz-Conca et al., 2022). In addition, CoQ10 activates ATP synthesis and mitigates mitochondrial ROS-induced oxidative damage (Rodriguez-Varela and Labarta, 2021). Particularly in older women, CoQ10 improves IVF/IVM success rates by restoring Krebs cycle activity, balancing ROS levels, and reducing DNA damage and oocyte apoptosis (Brown and McCarthy, 2023). Interestingly, in cattle, vitamin E has been shown to outperform CoQ10 and vitamin C in supporting IVM, IVF, and embryo development under heat stress, as evidenced by elevated trophectoderm, ICM, and blastocyst cell counts (Maddahi et al., 2024).

In addition to these supplements, salidroside has been found to reduce ROS levels and enhance intracellular glutathione (GSH) concentrations, promoting cytoplasmic maturation via increased ATP production, mitochondrial membrane potential, and mtDNA copy number. Moreover, salidroside activates MAPK phosphorylation, driving nuclear oocyte maturation and blastocyst pluripotency (Shi et al., 2023). Similarly, α-Ketoglutarate (α-KG), a TCA cycle metabolite, enhances follicle numbers and oocyte quality in aging oocytes (Wang H. et al., 2023). Nicotinamide mononucleotide (NMN) supplementation also restores mitochondrial function, suppresses ROS and apoptosis, and improves ART outcomes (Miao et al., 2020). Importantly, vitamin D supplementation has been shown to improve embryo quality in ICSI procedures by elevating follicular fluid and serum vitamin D levels (Baldini et al., 2024).

Finally, folic acid has been observed to influence *in vitro* oocyte maturation and gene expression patterns (Gennari et al., 2021), while asiatic acid reduces oxidative stress by boosting GSH production, ATP generation, and mitochondrial membrane potential (Qi et al., 2021). In a similar vein, anethole elevates ferric-reducing antioxidant power (FRAP), cleavage/morula/blastocyst rates, and mitochondrial membrane potential, all of which contribute to improved embryo development (Sa et al., 2020). These findings collectively underscore the significant role of dietary supplements in enhancing mitochondrial function and oocyte quality, thereby offering potential therapeutic strategies for improving ART outcomes.

### 2.2 Antioxidant activity

Oxidative stress, which is a major contributor to oocyte aging, is mitigated by antioxidant supplements that operate downstream of mitochondrial dysfunction by directly neutralizing oxidative damage through exogenous radical scavenging and endogenous defense potentiation, thereby enhancing cellular redox balance and preserving oocyte genomic integrity and developmental competence (Lucia Dos Santos Silva et al., 2023). For instance, in rabbit models, a quercetin-supplemented diet positively correlates with retrieved oocyte number, follicle count, and the proportion of A-grade oocytes; however, it does not significantly affect oocyte maturation (Naseer et al., 2017). Similarly, in mice, *Euterpe oleracea* exhibits variable effects on antioxidant pathways and cell growth by upregulating β-adrenergic signaling while downregulating apoptosis and proinflammatory signaling (Katz-Jaffe et al., 2020). Furthermore, melatonin plays a critical role in protecting against nonylphenol-induced oxidative stress and DNA damage in mice by rescuing mitochondrial membrane potential and correcting aberrant mitochondrial distribution via lysosomal regulation through Rab11 and lysosomal-associated membrane protein 2 (LAMP2) (Hu et al., 2022). Additionally, melatonin improves IVF outcomes by upregulating ATPase copper-transporting beta (ATP7B) and glutathione peroxidase 4 (GPX4) gene expression, thereby enhancing resilience against metal-induced toxicity and oxidative stress. *In vitro* studies using HGL5 cells, melatonin has been demonstrated to restore glycolysis, tricarboxylic acid (TCA) cycle activity, and redox balance, effectively protecting oocytes (Tsui et al., 2024).

In addition to melatonin, boric acid has been shown to preserve ovarian reserve by restoring stem cell factor (SCF) and AMH levels, downregulating sirtuin 1 (SIRT1), and upregulating mTOR signaling. Consequently, boric acid reduces CAT and SOD activity while suppressing MDA, tumor necrosis factor-alpha (TNF-α), interleukin-6 (IL-6), and neutrophil protease (NP) levels (Onder et al., 2023). Moreover, L-carnitine, when combined with bone marrow mesenchymal stem cell-conditioned medium, enhances IVF rates by modulating endometriosis-induced nitro-oxidative stress, increasing TAC, reducing NO levels, and correlating with improved blastocyst formation (Kalehoei et al., 2022). Silibinin also counteracts butyl benzyl phthalate toxicity by reducing autophagy and oxidative stress (Li Y. et al., 2023). Notably, zearalenone (ZEN) disrupts oocyte-cumulus cell interactions, delays cell cycle progression, and induces cytoskeletal abnormalities; however, these effects are mitigated by ZEN modification with hydrated sodium calcium aluminosilicate (Xu et al., 2021).

The beneficial effects of supplementing n-3 polyunsaturated fatty acids (PUFAs) on cow reproduction have been previously reported. Freret et al. confirmed that supplementation with n-3 PUFAs modify lipid composition in oocyte membranes, enhancing membrane fluidity and reducing susceptibility to lipid peroxidation (Freret et al., 2019). Alpha-lipoic acid (ALA) supplementation has been shown to reverse cyclophosphamide (CY)-induced meiotic maturation failure in oocytes. CY disrupts cytoskeletal assembly, organelle dynamics, and cortical granule/mitochondrial integrity—key indicators of cytoplasmic maturation. Remarkably, ALA suppresses oxidative stress-induced apoptosis and DNA damage, thereby protecting oocytes from CY-induced deterioration (Wang W. et al., 2023). In sheep models, zinc reduces ROS levels and enhances mitochondrial activity and GSH concentrations, thereby improving oocyte quality and embryonic development during *in vitro* maturation (IVM) (Yao et al., 2023). Similarly, zinc chloride (ZnCl2) and sodium selenite (Na2SeO3) promote oocyte progression to metaphase II during IVM, increasing TAC and reducing hydrogen peroxide (H_2_O_2_) and MDA levels, which correlate with upregulated antiapoptotic and antioxidative gene expression (Khalil et al., 2021). In contrast, lycopene scavenges oocyte ROS and enhances embryo cleavage under heat shock conditions, increasing inner cell mass (ICM) numbers without significantly affecting blastocyst development (Residiwati et al., 2021). These findings collectively highlight the diverse mechanisms by which antioxidants can mitigate oxidative stress and improve oocyte quality, offering promising therapeutic avenues for enhancing reproductive outcomes.

### 2.3 Epigenetically regulated metabolic pathways

Epigenetically regulated metabolic pathways play a pivotal role in enhancing oocyte quality and developmental competence. Notably, fibroblast growth factor 2 (FGF2), leukemia inhibitory factor (LIF), and insulin-like growth factor 1 (IGF1), collectively termed FLI medium, significantly improve mouse oocyte quality, as evidenced by enhanced blastocyst formation rates. Specifically, FLI medium promotes glucose metabolism through the pentose phosphate pathway, hexosamine biosynthesis, and glycolysis, while also upregulating transcripts of endothelial growth factor-like factors, reducing spindle abnormalities, and enhancing cumulus cell expansion. During IVM, FLI medium activates key signaling pathways, including the phosphorylation of protein kinase B (AKT), mitogen-activated protein kinase 1/3 (MAPK1/3), signal transducer and activator of transcription 3 (STAT3), and the mammalian target of rapamycin (mTOR) downstream target ribosomal protein S6 kinase B1 (RPS6KB1) (Nahar et al., 2024). Intriguingly, FLI medium, when combined with porcine follicular fluid, methionine, and cysteine supplementation, correlates with reduced polyspermic zygote formation and increased monospermic zygote formation (Currin et al., 2022). Additionally, L-carnitine modulates gene expression during embryo development (Carrillo-G et al., 2023), while alanine enhances embryonic competence by upregulating fibroblast growth factor receptor 2 (FGFR2) and POU class 5 homeobox 1 (POU5F1) mRNA levels, as well as increasing intra-oocyte GSH concentrations (Lee et al., 2019).

Other interventions further underscore the importance of metabolic regulation in oocyte development. For instance, the cathepsin B inhibitor E-64, when added to IVM medium, improves the developmental competence of ovum pick-up-derived immature oocytes (Balboula et al., 2022). Similarly, granulocyte colony-stimulating factor (G-CSF) enhances IVM of poor-quality cumulus-oocyte complexes (Cai et al., 2024). Interestingly, introducing mitochondrial DNA (mtDNA) into *Sus scrofa* oocytes alters DNA methylation profiles and gene expression, thereby modifying epigenetic programming during oogenesis (Okada et al., 2022). mtDNA supplementation studies reveal upregulated blastocyst-related genes, addressing mtDNA deficiency during pregnancy (Oka et al., 2023). Furthermore, myo-inositol plays a critical role in female endocrine and metabolic balance. Beyond its antioxidant activity, myo-inositol improves oocyte quality, enhances fertilization rates, and supports early embryonic development by modulating insulin signaling and FSH sensitivity (Alviggi et al., 2016). Recent evidence highlights that myo-inositol, administered with D-chiro-inositol in a physiological ratio of 40:1, which synergistically improves ovarian function and metabolic parameters in patients with PCOS (Dinicola et al., 2021). The efficacy of myo-inositol in improving IVF outcomes is further supported by clinical trials. For instance, supplementation with 1 g myo-inositol and 400 µg folic acid significantly increased mature oocytes in 133 PCOS women (Vartanyan et al., 2017), while 4 g myo-inositol and 400 µg folic acid reduced rFSH dose and cycle duration while increasing pregnancy rates in 98 infertile PCOS patients (Emekci Ozay et al., 2017).

Growth hormone (GH) supplementation has also been shown to increase euploid blastocyst rates, potentially reducing aneuploidy and improving pregnancy outcomes in cases of recurrent pregnancy loss (Guo et al., 2023). Additionally, GH lowers cycle cancellation rates, improves endometrial patterns, and enhances implantation and pregnancy rates (Lan et al., 2019). Similarly, putrescine increases meiosis resumption rates, oocyte cleavage efficiency, and blastocyst cell counts (Bicici et al., 2023). Propylene glycol alters gene expression and follicular fluid composition (Gamarra et al., 2018), while cytokine supplementation improves somatic cell nuclear transfer (SCNT) efficiency in IVF (Keim et al., 2023). Importantly, dietary interventions targeting oocyte quality are guided by follicular fluid fatty acid composition analyses, which reveal elevated levels of eicosapentaenoic acid (EPA) and docosahexaenoic acid (DHA) (Kermack et al., 2021). Probiotics and prebiotics modulate gut microbiota activity through amino acid metabolism, thereby mitigating metabolic syndrome and supporting female reproductive health (Dai et al., 2015). Collectively, these findings highlight the intricate interplay between epigenetic regulation, metabolic pathways, and oocyte quality, offering promising strategies to optimize reproductive outcomes Table 1.

**TABLE 1:** Summary of dietary supplements, mechanisms, and effects on oocyte quality.

Supplement	Mechanism	Effect on oocyte quality	References
Nicotinamide Riboside (NR)	Enhances mitochondrial ATP synthesis, reduces ROS, prevents DNA damage	Restores MII oocyte quality, improves chromosomal integrity	Li H. et al. (2023), Zhu et al. (2025)
CoQ10	Activates ATP synthesis, reduces oxidative stress, modulates glucocorticoid receptor	Improves IVF/IVM success rates, supports oocyte maturation	Ruiz-Conca et al. (2022), Rodriguez-Varela and Labarta (2021), Brown and McCarthy (2023)
Vitamin E	Scavenges ROS, enhances membrane stability	Supports IVM, IVF, and embryo development under heat stress	Maddahi et al. (2024)
Salidroside	Increases ATP production, enhances mitochondrial membrane potential	Promotes cytoplasmic and nuclear maturation, improves blastocyst pluripotency	Shi et al. (2023)
α-Ketoglutarate (α-KG)	Enhances TCA cycle activity, reduces oxidative stress	Improves follicle numbers and oocyte quality in aging oocytes	Wang H. et al. (2023)
Nicotinamide Mononucleotide (NMN)	Restores mitochondrial function, suppresses ROS and apoptosis	Improves ART outcomes	Miao et al. (2020)
Vitamin D	Elevates follicular fluid and serum vitamin D levels	Enhances embryo quality in ICSI procedures	Baldini et al. (2024)
Folic Acid	Modulates gene expression, supports DNA synthesis	Improves *in vitro* oocyte maturation	Gennari et al. (2021)
Asiatic Acid	Boosts GSH production, enhances ATP generation	Reduces oxidative stress, improves mitochondrial function	Qi et al. (2021)
Anethole	Elevates FRAP, enhances mitochondrial membrane potential	Improves cleavage, morula, and blastocyst rates	Sa et al. (2020)
Quercetin	Neutralizes oxidative stress, upregulates β-adrenergic signaling	Increases retrieved oocyte number and follicle count	Naseer et al. (2017)
Melatonin	Protects against oxidative stress, rescues mitochondrial membrane potential	Improves IVF outcomes, protects against nonylphenol-induced damage	Hu et al. (2022), Tsui et al. (2024)
Boric Acid	Restores SCF and AMH levels, modulates mTOR signaling	Preserves ovarian reserve, reduces oxidative stress	Onder et al. (2023)
L-Carnitine	Modulates nitro-oxidative stress, enhances TAC	Improves IVF rates and blastocyst formation	Kalehoei et al. (2022)
Silibinin	Reduces autophagy and oxidative stress	Counteracts butyl benzyl phthalate toxicity	Li Y. et al. (2023)
n-3 PUFAs	Modifies lipid composition, reduces lipid peroxidation	Enhances membrane fluidity, improves oocyte quality	Freret et al. (2019)
Alpha-Lipoic Acid (ALA)	Suppresses oxidative stress-induced apoptosis, protects against DNA damage	Reverses cyclophosphamide-induced meiotic maturation failure	Wang W. et al. (2023)
Zinc	Reduces ROS, enhances mitochondrial activity	Improves oocyte quality and embryonic development during IVM	Yao et al. (2023), Khalil et al. (2021)
Lycopene	Scavenges ROS, enhances embryo cleavage	Improves inner cell mass (ICM) numbers under heat shock conditions	Residiwati et al. (2021)
FLI Medium (FGF2, LIF, IGF1)	Promotes glucose metabolism, upregulates key signaling pathways	Enhances blastocyst formation, reduces spindle abnormalities	Nahar et al. (2024), Currin et al. (2022)
Myo-Inositol	Modulates insulin signaling, improves FSH sensitivity	Enhances oocyte quality, fertilization rates, and early embryonic development	Alviggi et al. (2016), Dinicola et al. (2021)
Growth Hormone (GH)	Reduces aneuploidy, improves endometrial patterns	Increases euploid blastocyst rates, enhances implantation and pregnancy rates	Guo et al. (2023), Lan et al. (2019)
Putrescine	Enhances meiosis resumption, improves blastocyst cell counts	Increases oocyte cleavage efficiency	Bicici et al. (2023)

## 3 Human clinical trials on dietary supplements and oocyte outcomes

In human clinical trials, dietary interventions with methyl donors during FSH stimulation in women with PCOS have demonstrated significant benefits. Specifically, these interventions reduce FSH requirements and improve implantation rates, while follicular homocysteine levels are negatively correlated with clinical pregnancy rates. These findings suggest that methyl donor-enriched diets may enhance outcomes in PCOS patients (Kucuk et al., 2023). Similarly, flaxseed oil supplementation in women with diminished ovarian reserve (DOR) has been shown to reduce recombinant human follicle-stimulating hormone (r-hFSH) dosage and stimulation duration, while increasing peak estradiol (E2), MII oocyte rate, fertilization, cleavage, and high-quality embryo rates. This indicates a positive correlation between flaxseed oil supplementation and improved MII oocyte rates in DOR patients (Chu et al., 2024). Furthermore, dehydroepiandrosterone (DHEA) supplementation in aging women has been found to increase day 3 embryo yield and top-quality day 3 embryos, as well as improve ongoing pregnancy rates and clinical pregnancy rates. These results suggest that DHEA enhances IVF outcomes in aging women, partially through the reprogramming of metabolic pathways (Li et al., 2021).

On the other hand, not all dietary interventions yield positive results. For instance, antioxidant supplementation in women with unexplained subfertility has not shown significant changes in age, BMI, basal FSH, mature MII oocyte count, or clinical pregnancy rate. This indicates that oral multivitamin/mineral antioxidants do not improve oocyte quality (Youssef et al., 2015). However, certain combination therapies have shown promise. Melatonin and myo-inositol combination therapy in patients with PCOS has demonstrated synergistic enhancement of oocyte quality and embryo quality, suggesting that this combined therapy is recommended for IVF protocols in PCOS (Pacchiarotti et al., 2016). Additionally, vitamin E and D3 co-supplementation in patients with PCOS has been found to increase clinical pregnancy and implantation rates while decreasing serum TAC. Although there is a weak association between vitamin D and implantation rate, these findings indicate that vitamins E/D3 improve IVF success via antioxidant mechanisms (Fatemi et al., 2017).

Moreover, n-3 PUFAs intervention in reproductive-age women has shown reduced omega-6/omega-3 ratio, FSH, and FSH response to GnRH, as well as decreased serum IL-1β/TNF-α in the obese subgroup. This suggests that omega-3 PUFA benefits women with diminished ovarian reserve (Al-Safi et al., 2016). In PCOS patients undergoing IVF, n-3 PUFA specifically improves oocyte maturation by inhibiting oxidative stress, clearing free radicals, and maintaining spindle/chromosome integrity (Ma et al., 2023). Furthermore, myo-inositol supplementation in poor responders undergoing ICSI has not shown differences in gonadotropin dose, oxidative stress index (OSI), or total retrieved and mature oocytes count. However, ovulation triggering improves fertilization rates and embryo quality (Nazari et al., 2020). Standardized multi-nutrient supplementation in women undergoing ICSI has been shown to improve pregnancy outcomes, indicating that multi-nutrient regimens enhance embryo developmental competence (Nouri et al., 2017). Multi-nutrient supplementation also confers positive effects on follicular output rate while reducing gonadotropin requirements (Gopinath et al., 2024). Additionally, early-onset estrogen supplementation improves the quality of retrieved immature oocytes, enhancing maturation rates during IVM cycles (Hatirnaz et al., 2021) Figure 1.

**FIGURE 1:**
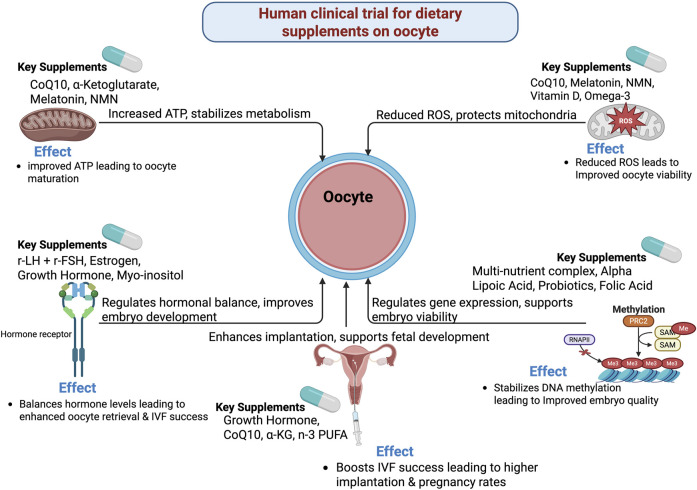
Mechanistic and biological pathways on human clinical trial for dietary supplements on oocyte.

## 4 Precision medicine in oocyte enhancement

The integration of precision medicine into reproductive health offers transformative potential for addressing female infertility, particularly through targeting the enhancement of oocyte quality. Precision medicine customizes interventions based on genetic, metabolic, and biomarker profiles, addressing the molecular heterogeneity that underlying infertility. Although the efficacy of dietary supplements remains debated, they have emerged as critical modulators of oocyte health, exerting their effects through antioxidant, mitochondrial, and hormonal pathways.

### 4.1 Genetic strategies

Genetic variability significantly influences ovarian reserve, oxidative stress responses, and mitochondrial function, thereby shaping individual responses to supplementation. For instance, polymorphisms in genes such as SOD or GPX4 can influence the efficacy of antioxidant therapies. Melatonin, which upregulates the expression of ATP7B and GPX4 in cumulus cells, may be particularly beneficial individuals with genetic variants that disrupt metal ion homeostasis or lipid peroxidation defenses (Tsui et al., 2024). Similarly, CoQ10’s ability to restore mitochondrial electron transport chain activity may be critical for carriers of PDSS2 or COQ6 mutations linked to age-related oocyte decline (Ben-Meir et al., 2015; Desbats et al., 2015).

In PCOS, INSR variants correlate with insulin resistance, guiding the use of myo-inositol to improve FSH signaling and glucose uptake (Unfer et al., 2017; Han et al., 2024). Genetic screening for such variants could stratify patients for personalized supplementation protocols, thereby enhancing ART outcomes.

### 4.2 Metabolic profiling

Metabolic dysregulation, including mitochondrial dysfunction or insulin resistance, significantly influences the efficacy of supplements. NAD + precursors such as NR can mitigate age-related NAD + depletion in oocytes, thereby restoring chromosomal integrity and mitochondrial membrane potential in murine models (Li H. et al., 2023). Women with metabolic syndrome or PCOS, often exhibiting mitochondrial inefficiency, may benefit from NMN to boost ATP production and reduce ROS (Miao et al., 2020). Lipidomic profiling of follicular fluid reveals elevated EPA and DHA correlating with improved oocyte maturation, particularly in PCOS patients (Freret et al., 2019; Ma et al., 2023). Precision lipid supplementation could thus target individuals with suboptimal fatty acid profiles. Similarly, α-KG, a TCA cycle intermediate, enhances follicle numbers in aging oocytes by restoring metabolic flux, suggesting utility in women with diminished ovarian reserve (Wang H. et al., 2023).

### 4.3 Biomarker-driven optimization

Biomarkers such as AMH, oxidative stress markers, and follicular fluid composition provide a means for real-time monitoring of interventions. CoQ10 supplementation has been shown to increase AMH-positive follicles and reduces oxidative DNA damage in cisplatin-treated rats, highlighting its role in preserving ovarian reserve (Ozcan et al., 2016). In humans, follicular fluid vitamin D levels correlate with embryo quality, supporting its use in vitamin D-deficient patients undergoing ICSI (Baldini et al., 2024).

Dynamic biomarkers, such as mitochondrial membrane potential or redox balance (GSH/GSSG ratio), could guide dose adjustments. For example, melatonin’s restoration of glycolysis and TCA cycle activity in HGL5 granulosa cells suggests that women with metabolic dysregulation in cumulus-oocyte complexes may require higher doses (Tsui et al., 2024). Similarly, elevated FDX1 levels linked to mitochondrial efficiency could identify candidates for CoQ10 or ubiquinol supplementation (Serrano et al., 2022).

### 4.4 Challenges and future directions

Although significant progress has been made, key challenges remain, including genetic heterogeneity such as *MTHFR* variants complicating folate dosing, limited clinical validation of oxidative stress biomarkers for infertility, and reliance on animal models, highlighting the need for human RCTs with stratified cohorts (Vitagliano et al., 2021; Woodward et al., 2022). Emerging tools such as multi-omics (genomics, metabolomics) and AI-driven predictive models offer solutions for instance, integrating genomic data with follicular fluid metabolomics to predict antioxidant responses (astaxanthin and vitamin E) (Maddahi et al., 2024; Tripathi et al., 2023). Precision medicine is reshaping oocyte enhancement by aligning interventions like melatonin and CoQ10 (targeting mitochondrial dysfunction and oxidative stress in age-related decline) or myo-inositol and omega-3 PUFAs (addressing PCOS-specific dysregulation) with individual genetic, metabolic, and biomarker profiles. To realize this potential, future research must prioritize human trials, biomarker validation, and integrative omics approaches, advancing fertility care from empirical practices to evidence-based precision therapeutics.

## 5 Limitations of current evidence and recommendations for future research

The following section discusses the key limitations of existing studies on dietary interventions and oocyte quality, as well as actionable recommendations to address these gaps and advance future research. While the cited articles provide valuable insights, their mechanistic and clinical heterogeneities highlight the need for a more standardized and pathway-centric approach in this field.

### 5.1 Limitations

#### 5.1.1 Mechanistic gaps

One of the primary obstacles in optimizing dietary interventions for oocyte quality is the lack of clarity regarding nutrient-specific pathways. For instance, while quercetin has been linked to improved IVF oocyte retrieval outcomes, its apparent negative impact on embryo quality remains mechanistically unclear, which complicates its clinical application (Baltazar et al., 2018). Similarly, cysteamine’s dual role in upregulating molecular markers during cumulus-oocyte complex (COC) maturation while downregulating embryonic development-related genes highlights a knowledge gap in its gene regulatory networks (Doroftei et al., 2024). The role of α-KG in oocyte quality and stem cell dynamics despite its documented influence on differentiation and reprogramming lacks clarity, particularly in how these processes directly translate to reproductive performance (Karayiannis et al., 2018). Oxidative stress interventions also face mechanistic ambiguities: GSH only partially rescues ovarian follicle loss, suggesting incomplete understanding of redox balance in follicular survival (Hohos et al., 2020), while melatonin’s ability to ameliorate age-related meiotic defects via the SIRT2-dependent H4K16 deacetylation pathway requires deeper exploration to define its broader applicability (Wu et al., 2024). Metabolic interactions further exemplify mechanistic uncertainties. High-energy diets, though promoting follicular growth through elevated insulin, paradoxically impair oocyte quality, indicating unresolved links between metabolic signaling and oocyte competence (Li Z. et al., 2023). Similarly, fatty acid supplementation in bovine models fails to modify lipid metabolism or developmental capacity, underscoring a disconnect between fatty acid’s theoretical benefits and practical outcomes (Gardinal et al., 2018).

#### 5.1.2 Clinical heterogeneity

Clinical translation is hindered by variability in diagnostic and dosing frameworks. The absence of standardized criteria for ovarian dysfunction and “poor responder” classification limits generalizability across studies, complicating the identification of target populations (Chantrasiri et al., 2025). Dosing inconsistencies further exacerbate reproducibility challenges: melatonin’s broad dosage range (2–18 mg/day) and unclear optimal thresholds for selenium in female reproductive health reflect a lack of precision in therapeutic protocols (Genario et al., 2019; Chowdhury et al., 2021). Variable supplement durations, as seen in bovine studies where prepartum whole raw soybean supplementation showed no effect on oocyte quality, highlight the need for standardized timelines (Tomita et al., 2023). Species-specific differences also limit translational relevance. For example, melatonin’s integration into bovine breeding management faces practical challenges despite its theoretical benefits, and rodent-derived data on high-fat diets or fatty acid supplementation poorly predict human responses, emphasizing the need for human-centric models (Piscopo et al., 2025). Even promising interventions, like sunflower seed supplementation or crude protein’s adverse effects on embryo development, lack consistency across species, underscoring the risks of extrapolating animal data to clinical practice (Plante-Dube et al., 2021; Lim et al., 2020).

### 5.2 Recommendations

#### 5.2.1 Pathway-centric trials

Mitochondrial and Redox Pathways: Targeted interventions aimed at enhancing mitochondrial function and redox balance are critical for improving oocyte quality. The evaluation of compounds such as CoQ10, DHEA, and Cleo-20 T3 could potentially increase the levels of ferredoxin 1 (FDX1), a key regulator of mitochondrial TCA cycle activity and electron transport chain efficiency, thereby mitigating lipid peroxidation and apoptosis in oocytes (Serrano et al., 2022). FDX1’s dual role in energy metabolism and oxidative stress reduction positions it as a therapeutic target for age-related declines in oocyte competence, particularly in IVF settings where mitochondrial dysfunction is prevalent.

##### 5.2.1.1 Kinase signaling

Evaluating kinase-driven pathways offers opportunities to address metabolic and maturation defects in oocytes. For instance, combining myo-inositol with folic acid in PCOS patients modulates extracellular signal-regulated kinase 1/2 (ERK1/2) phosphorylation, reducing phosphorylated AKT levels and improving insulin sensitivity, a key factor in PCOS-associated infertility (Unfer et al., 2017). Similarly, FLI medium in follicular fluid enhances oocyte meiotic maturation by activating MAPK pathways, which regulate cytoskeletal dynamics and developmental competence (Thongkittidilok et al., 2022). Prioritizing trials on kinase signaling could refine therapies for conditions like PCOS and poor ovarian response.

##### 5.2.1.2 Antioxidant synergy

Optimizing antioxidant combinations, such as chlorogenic acid, curcumin, and β-mercaptoethanol, may amplify protection against oxidative damage during embryo development. While individual antioxidants show partial efficacy, synergistic formulations could enhance blastocyst formation rates by targeting multiple redox pathways simultaneously (Anchordoquy et al., 2022). This approach is particularly relevant for patients with elevated oxidative stress, such as those with advanced maternal age or metabolic disorders.

#### 5.2.2 Standardized dosing

##### 5.2.2.1 Reproductive protocols

Defining optimal dosages for supplements like melatonin (2–18 mg/day) is essential to balance efficacy and safety, especially regarding gestational and neonatal outcomes (Chowdhury et al., 2021). Trace minerals (Cu, Se, Mn, Zn) during *in vitro* maturation (IVM) improve embryo quality, but standardized thresholds are needed to avoid toxicity and ensure reproducibility (Tabatabaie et al., 2022). Similarly, omega-3 PUFAs require dose-dependent studies to validate their role in restoring ovarian gene expression disrupted by high-fat diets (Li et al., 2020).

##### 5.2.2.2 Dietary interventions

Standardizing components of the Mediterranean diet rich in antioxidants, monounsaturated fats, and polyphenols could improve IVF pregnancy rates by reducing inflammation and oxidative stress (Machado et al., 2020). Concurrently, validating peripartum oleic acid dosing is critical to support follicular and oocyte health during metabolic stressors like negative energy balance (Mintziori et al., 2020).

##### 5.2.2.3 Toxicity thresholds

Establishing safe upper limits for supplements is vital. For example, crude protein over-supplementation may impair embryo development, necessitating guidelines to avoid unintended harm (Lim et al., 2020). Similarly, while niacin reduces oxidative stress, its optimal dosing for ovarian follicle rescue without adverse effects remains undefined (Almubarak et al., 2021). Prioritizing pathway-centric trials and standardized dosing frameworks will bridge mechanistic knowledge gaps and clinical inconsistencies, enabling precision-based strategies to enhance oocyte quality and fertility outcomes.

## 6 Conclusion

Dietary supplements and nutritional interventions influence oocyte quality through diverse mechanisms across species, targeting oxidative stress, mitochondrial function, and metabolic pathways. Key findings include the benefits of antioxidants in reducing ROS and improving IVF outcomes, the role of myo-inositol and n-3 PUFAs in enhancing oocyte quality in patients with PCOS, and the ability of CoQ10 and α-KG to counteract age-related oocyte decline. Preclinical studies highlight promising compounds such as quercetin, SDF-1, and lycopene, which improve oocyte retrieval, cytoplasmic maturation, and stress resilience in rodents and bovines. However, several limitations hinder translational progress, including inconsistent dosages, mixed outcomes, and a lack of standardized protocols. High-energy diets and crude protein supplementations may impair oocyte quality, while clinical heterogeneity and species-specific responses further complicate the application of preclinical findings. TO address these challenges, future research should prioritize human-centric studies, standardized dosing frameworks, and pathway-centric trials targeting mitochondrial function and antioxidant synergy. Establishing evidence-based protocols and focusing on clinical reproducibility will be essential to translate these insights into reliable therapies for infertility. In summary, while dietary supplements offer significant potential to improve oocyte quality, a more systematic and standardized approach is urgently needed to bridge the gap between preclinical discoveries and clinical applications.
